# Modular scaffolding by lncRNA HOXA10-AS promotes oral cancer progression

**DOI:** 10.1038/s41419-022-05071-6

**Published:** 2022-07-20

**Authors:** Yi-Tung Chen, Chia-Hua Kan, Hsuan Liu, Yu-Hao Liu, Chih-Ching Wu, Yu-Ping Kuo, Ian Yi-Feng Chang, Kai-Ping Chang, Jau-Song Yu, Bertrand Chin-Ming Tan

**Affiliations:** 1 Molecular Medicine Research Center, Chang Gung University, Taoyuan, 333 Taiwan; 2 Research Center for Emerging Viral Infections, Chang Gung University, Taoyuan, 333 Taiwan; 3 Department of Biomedical Sciences, College of Medicine, Chang Gung University, Taoyuan, 333 Taiwan; 4 Department of Cell and Molecular Biology, College of Medicine, Chang Gung University, Taoyuan, 333 Taiwan; 5 Graduate Institute of Biomedical Sciences, College of Medicine, Chang Gung University, Taoyuan, 333 Taiwan; 6 Division of Colon and Rectal Surgery, Lin-Kou Medical Center, Chang Gung Memorial Hospital, Taoyuan, 333 Taiwan; 7 Department of Medical Biotechnology and Laboratory Science, College of Medicine, Chang Gung University, Taoyuan, Taiwan; 8 Department of Otolaryngology-Head & Neck Surgery, Lin-Kou Medical Center, Chang Gung Memorial Hospital, Taoyuan, 333 Taiwan; 9 Department of Neurosurgery, Lin-Kou Medical Center, Chang Gung Memorial Hospital, Taoyuan, 333 Taiwan

**Keywords:** Cell growth, Oral cancer, Long non-coding RNAs

## Abstract

Recent findings have implicated long noncoding RNAs (lncRNAs) as pivotal gene regulators for diverse biological processes, despite their lack of protein-coding capabilities. Accumulating evidence suggests the significance of lncRNAs in mediating cell signaling pathways, especially those associated with tumorigenesis. Consequently, lncRNAs have emerged as novel functional regulators and indicators of cancer development and malignancy. Recent transcriptomic profiling has recognized a tumor-biased expressed lncRNA, the HOXA10-AS transcript, whose expression is associated with patient survival. Functional cell-based assays show that the HOXA10-AS transcript is essential in the regulation of oral cancer growth and metastasis. LncRNA expression is also associated with drug sensitivity. In this study, we identify that HOXA10-AS serves as a modular scaffold for TP63 mRNA processing and that such involvement regulates cancer growth. These findings provide a functional interpretation of lncRNA-mediated molecular regulation, highlighting the significance of the lncRNA transcriptome in cancer biology.

## Introduction

The predominant constituents of the transcriptome are noncoding nucleic acids, including long, noncoding RNAs (lncRNAs) and microRNAs [1]. Despite lacking protein-coding potential, these have emerged as important molecular players in gene regulation and biological switches in various physiological and pathological processes, particularly lncRNAs [2]. During gene transcription, lncRNAs can guide or provide a scaffold for the recruitment of chromatin-modifying factors that fine-tune transcription initiation. At the posttranscriptional level, lncRNAs can bind to target RNAs and alter their structural stability and splicing patterns. Additionally, lncRNAs are reportedly to interact with miRNAs and sponge their endogenous activities [3–5]. These diverse lncRNA-mediated regulatory mechanisms emphasize their significance and functional relevance in shaping the transcriptome and the resulting biological consequences.

In the field of cancer research, lncRNAs exhibit unique expression profiles in various human cancers that provide strong correlations with disease progression. As a result, their collective clinical value in predicting the patient outcome is significant [6–10]. For example, lncRNA MALAT1 has been implicated as an oncogenic gene in numerous cancers; in particular, its regulation of oral cancer progression has been functionally characterized [11–14]. On the other hand, lncRNAs can function as tumor suppressors, such as lncRNA-p21, which inhibits JAK2/STAT3 signal activation and represses STAT3-induced oncogenic potential in head and neck carcinoma [15]. Consequently, dysregulated expression of lncRNAs has been functionally linked to cancer development and progression. Investigation of these noncoding regulators may shed new light on tumorigenesis and malignancy that possibly provide new avenues for therapeutic interventions [16–18].

In this study, we recognized an oncogenic lncRNA, HOXA10-AS transcript, from transcriptomic profiling. Systematic functional assays uncovered a central role of the HOXA10-AS molecule in oral cancer growth, metastasis, and cell survival. Next, we identified HOXA10-AS lncRNA scaffolding UPF1 protein binding to the TP63 transcript for subsequent processing. Such regulation determines the biological consequences of growth regulation. These findings illustrate that lncRNAs modify processing mechanisms to regulate gene expression, providing a functional interpretation for their roles in tumorigenesis and cancer progression.

## Materials and methods

### RNA extraction, reverse transcription, and quantitative PCR (RT-qPCR)

Total RNA was extracted by TRIzol reagent (Invitrogen), and reverse transcribed into complementary DNA (cDNA) by MML-V reverse transcriptase (Invitrogen) with random hexamers. Individual gene expression was analyzed by real-time quantitative PCR (iQ5 Gradient Real-Time SYBR-Green PCR system) with specific primers and analyzed by CFX Manager Software (Bio-Rad, CA, USA), and the sequences of used primer were listed in Table S1. Relative gene expression was determined and calculated by the delta Ct method, and all results were obtained from at least three independent experiments.

### Statistical analysis

All experiments were performed with at least three independent experiments, and the number of replicates was annotated in the corresponding legends. Statistical significance was assessed by Student’s *t*-test, and presented in *p* value form in this figures: ns *p* > 0.05, *p < 0.05, **p < 0.01, ***p < 0.001.

### Plasmids construction for gene knockdown and overexpression

RNAi-mediated gene silencing was performed by using the pLKO-TRC017 RNAi system, with the target sequences annealed and ligated into the TRC017 vectors. For constructing the HOXA10-AS expression vector, the sequences were amplified from the cDNA sample with designed primers by PCR assay. The resulting PCR products were ligated into the cloning vector with HE Swift Cloning Kit (BIOTOOLS), and subsequently sub-cloned into the expression vector pcDNA3.1 (−) and lentiviral pLAS3W vector. All used primers in this work were listed in Table S1. Constructed plasmids were packaged into viral particles and infected into cells, and experimental procedures were conducted based on the manufacturer’s instructions (RNAi core, Academia Sinica, Taiwan).

### Cell culture

SAS cells were cultured in high-glucose Dulbecco’s modified Eagle’s medium, 1× NEAA was added for OECM-1 cell culture. SCC25 cells were cultured in Dulbecco’s Modified Eagle Medium: Nutrient Mixture F-12 containing 1× NEAA, 1 mM sodium pyruvate, and 400 ng/mL hydrocortisone. All culture media were supplemented with 10% heat-inactivated fetal bovine serum and 1 U/mL penicillin-streptomycin, and all reagents were purchased from Thermo Fisher Scientific. Cells were maintained at 37 °C with 5% CO_2_ in a humidified incubator.

### MTT cell proliferation assay and colony formation assay

For the MTT proliferation assay, 2.5 × 10^4^ cells were seeded in a 24-well culture plate and incubated with MTT reagent (Sigma-Aldrich) for 1 h, and formed precipitates were dissolved and quantified by spectrophotometry at 570 nm for determining cell viability. In colony formation assay, 2.5 × 10^3^ cells were seeded in a six-well plate for a 7-day culture, and the forming colonies were stained by crystal violet and quantified by ImageJ software.

### In vivo mouse xenograft experiment

NOD.CB17-Prkdc^scid^/JNarl male mice (6 weeks old) were provided by the National Laboratory Animal Center (NLAC). Specific cancer cell lines were collected (1 × 10^6^ cells) and injected subcutaneously into rear flank of mice with a 26-gauge needle. Tumor formation and growth curves were monitored by a Vernier caliper at the indicated time-points, and tumor volumes (mm^3^) were calculated with the formula: length × width^2^ × 0.52. Tumor weight was measured after mice sacrifice. The animal experiment was approved by Laboratory Animal Center, Chang Gung University.

### Wound-healing assay and Transwell experiments

For the wound-healing assay, 1.5 × 10^6^ cells were seeded in a six-well plate and subsequently scratched by a 20 μL pipette tip. Wounded cell migration at the indicated time-point was recorded via the Cytation^TM^ 5 Cell Imaging instrument. Transwell migration and invasion assays were performed with Transwell Polystyrene Membrane Insert (Corning) and Matrigel (BD Biosciences). Briefly, 1 × 10^5^ cells were seeded into the Transwell chamber coated with Matrigel (invasion) or without (for migration); serum-free medium was added to the top of the chambers, and the lower level was filled with culture medium. After 16 h incubation, the migrating (invading) cells through chambers were fixed, crystal violet stained, and quantified by ImageJ software.

### RNA-immunoprecipitation (RNA-IP) experiment

RNA-IP assay was performed according to the previous report [19]. Briefly, cells were washed with PBS and lysed by Polysomal Lysis Buffer [100 mM KCl, 5 mM MgCl_2_, 10 mM HEPES (pH 7.0), 0.5% NP-40, 1 mM DTT, 50 U/ml RNaseOUT, and protease inhibitor], and the lysate was centrifuged at 12,000×*g* for 15 min at 4 °C. The supernatant was collected and incubated with control IgG or antibody-coated Dynabeads Protein G (Invitrogen) for 3 h at 4 °C. The precipitated complex was washed and treated with 10 U DNase I (Fermentas) at 37 °C for 15 min. TRIzol reagent was added for RNA extraction, and the precipitated RNA was reverse transcribed to cDNA, and subsequently analyzed by RT-qPCR assay. The used antibodies were listed as follows: control rabbit IgG (P120-101, Bethyl), UPF1 antibody (mAb #12040, Cell Signaling Technology), and Anti-Argonaute-2 antibody (ab32381, Abcam).

### Nuclear and cytoplasmic fractionation experiment

Experimental procedure was performed based on the previous work [20]. Briefly, cells were harvested and washed with PBS, and the cell lysate was centrifuged into a pellet. Nuclear fractionation buffer, containing 10 mM Tris-HCl (pH 7.8), 140 mM NaCl, 1.5 mM MgCl_2_, 0.5% NP-40, 3 U/ml RNaseOUT (Invitrogen), was added to resuspend cell pellet and incubated for 5 min at 4 °C. The lysates were subsequently centrifuged at 1000×*g* for 4 min, and the resultant supernatant and pellet were isolated respectively to serve as cytoplasmic and nuclear fractions. Total RNA from the fractions was extracted by TRIzol reagent, and subsequently, reverse transcribed to cDNA. U48 and 7SL RNA expression was analyzed and served as the indicators for nuclear and cytoplasmic compartments, respectively.

### RNA fluorescence in situ hybridization

The human TP63 gene sequence was amplified by PCR, and the amplicon was sub-cloned into an expression vector for probe synthesis. A specific probe against the TP63 gene was synthesized from the linearized plasmid by FISH Tag™ RNA Green Kit, with Alexa Fluor™ 488 dye (Invitrogen, CA, USA). The subsequent hybridization procedure was carried out according to the manufacturer’s instructions. Briefly, cells were rinsed, fixed, permeabilized, and subjected to proteinase K treatment. Probe hybridization was done with buffer (50% formamide, 5×SSC) for overnight at 55 °C. The hybridized sample was washed and counter-stained with Hoechst 33342 (Thermo Fisher Scientific) for nuclei. Visualization of samples was performed with ZEISS LSM780 confocal microscope.

## Results

### Identification of HOXA10-AS as a cancer-associated noncoding transcript

**Fig. 1:**
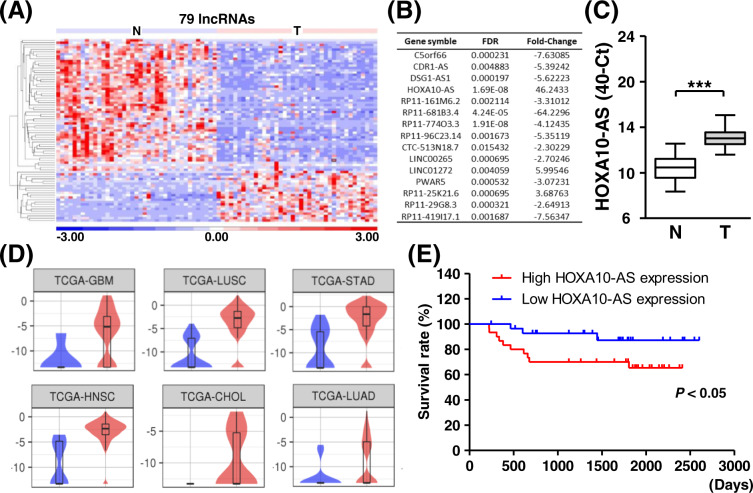
Identification of HOXA10-AS as a cancer-associated noncoding RNA. **A** Hierarchical clustering analysis of the differentially expressed lncRNAs in tumor tissues (T) and their adjacent normal sections (N) with OSCC sequencing is shown. **B** A list of the highly differential lncRNAs with their fold changes in expression and corresponding false discovery rate is presented. **C** HOXA10-AS expression in the paired normal and tumor tissues was measured by RT-qPCR assay and is presented as ΔCt method (*n* = 18). **D** Comparative expression profiles of HOXA10-AS transcript across different tumor types from the TCGA dataset are presented. **E** Kaplan–Meier survival curves of patients stratified according to HOXA10-AS expression are shown. The HOXA10-AS RNA expression of the clinical samples was measured and further ranked to assess the association between expression and the survival rate (*n* = 115).

For the exploration of lncRNAs in oral carcinoma, we utilized the transcriptome profiling of paired tumor tissues and adjacent normal sections [21]. Seventy-nine lncRNAs were found with differentiated expression and these were subjected to a hierarchical clustering analysis (Fig. 1A, B). One of the most notably upregulated lncRNAs in tumor sections with the lowest false discovery rate was the antisense transcript of the HOXA10 gene (termed HOXA10-AS). This transcript was, therefore, selected for further investigation. Independent RT-qPCR assay from other cohort specimens also showed the upregulation of HOXA10-AS transcript in tumor tissues compared to adjacent normal sections (Fig. 1C). This confirmed the data from next-generation sequencing. Similarly, integrating public TCGA datasets coordinately indicated HOXA10-AS tumor-specific expression among various cancers (Fig. 1D). Further, the association of HOXA10-AS expression with patient survival supported its significance in oral cancer progression (Fig. 1E). The clinical relevance of HOXA10-AS transcript was also observed in cervical and lung cancers [22] (Fig. S1A, B). RT-qPCR analysis showed a higher expression of HOXA10-AS in a malignant oral cancer cell line than in OC3 benign cells, implying its potential for oral cancer development (Fig. S1C). In terms of spatial distribution, the subcellular fraction experiment showed that HOXA10-AS was detectable in both nuclear and cytoplasmic compartments (Fig. S1D). This integrative analysis of public and in-house datasets reveals the fundamental role of the HOXA10-AS transcript in cancer biology.

### LncRNA HOXA10-AS promotes oral cancer growth

**Fig. 2:**
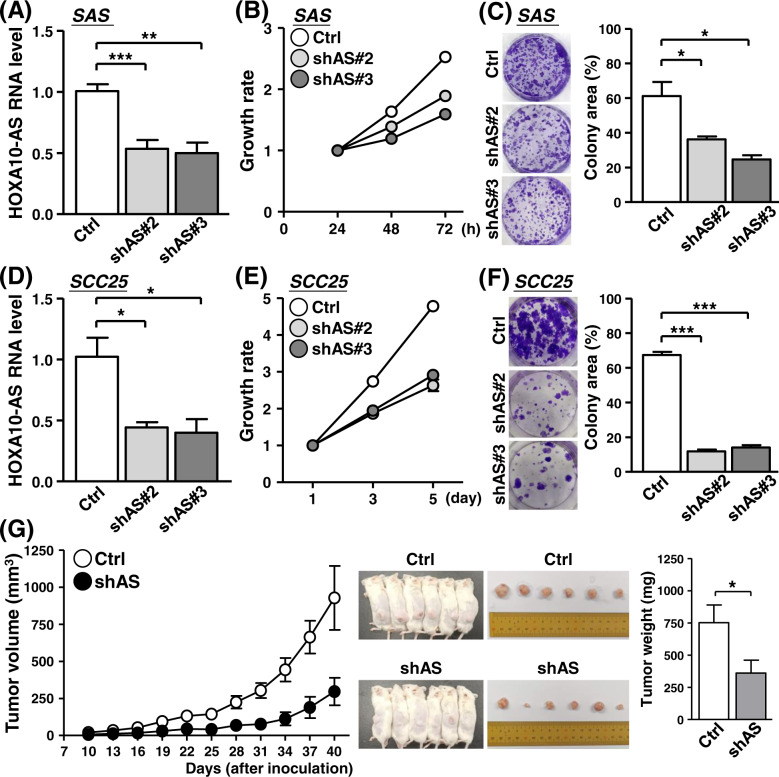
Requirement of the HOXA10-AS transcript for maintaining oral cancer cell growth. **A**–**F** Oral cancer lines SAS and SCC25 were subjected to HOXA10-AS knockdown by means of lentiviral infection (shAS#2 and shAS#3). Knockdown efficacy was assessed by RT-qPCR assay (*n* = 4) (**A**, **D**). The cell proliferation rate (**B**, **E**) and colony-forming ability (**C**, **F**) of knockdown cells were assessed by the MTT method and crystal violet staining (*n* = 4), respectively. Representative images of the colonies and quantified results based on colony area are shown. **G** Mouse xenograft experiments were performed by inoculating control and HOXA10-AS knockdown SAS cells (shAS). Tumors formed at the indicated time points were dissected and their volumes measured (left). The middle panel shows photographs of mice bearing tumors as well as surgically removed tumors. The bar graph depicts the average weights of the tumor masses dissected from the indicated groups at sacrifice.

Based on the association of HOXA10-AS with cancer progression, we examined its effect on cancer-associated phenotypes. RNAi-mediated silencing system was employed for HOXA10-AS knockdown, and the efficiency of the specific shRNAs was confirmed by RT-qPCR assay (Fig. 2A). We then performed proliferation assays and found reduced cell growth and clonogenicity in HOXA10-AS knockdown SAS cells (Fig. 2B, C). Likewise, targeting HOXA10-AS expression in another oral cancer cell line, SCC25 cells, led to the inhibition of cancer growth and colony formation (Fig. 2D–F). These results demonstrated the pro-growth role of the HOXA10-AS transcript in oral cancer cells. Next, the established HOXA10-AS knockdown cell lines were subcutaneously grafted into immunocompromised mice to evaluate in vivo tumor formation. Parameters including tumor volume, size, and weight were recorded and analyzed over a 6-week period for this mouse model. Consistent with cell-based experiments, the formed tumor volume in the HOXA10-AS knockdown group was significantly smaller than in the control group (Fig. 2G, left panel). The resected tumor weight was subsequently measured. The results supported the previous in vitro findings, as HOXA10-AS appeared to also play a crucial role in in vivo cancer growth (Fig. 2G, right panel). These findings demonstrated the pivotal role of HOXA10-AS in oral cancer development. Targeting HOXA10-AS expression caused a detrimental impact on oral cancer growth.

**Fig. 3:**
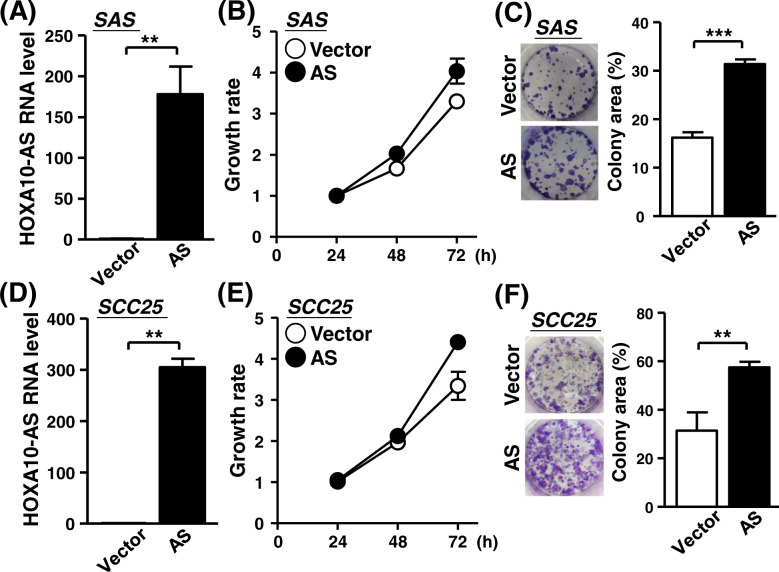
HOXA10-AS overexpression promoted cancer cell growth. **A**–**F** SAS and SCC25 cells were infected with control and HOXA10-AS overexpression constructs. HOXA10-AS expression of the infected cells was analyzed by RT-qPCR assay (*n* = 3) (**A**, **D**). The cell proliferation rate (**B**, **E**) and clonogenicity (**C**, **F**) of the cells were measured by the MTT method and crystal staining (*n* = 3), respectively.

To further explore the functional role of the HOXA10-AS transcript, we constructed a HOXA10-AS expression vector and delivered it into cells. The RT-qPCR assay showed ectopic HOXA10-AS overexpression of transfected cells, affirming the constructing efficiency (Fig. 3A). Cell proliferation and clonogenicity was moderately enhanced by HOXA10-AS overexpression, representing the reverse phenotype to our knockdown experiments (Fig. 3B, C). Incidentally, HOXA10-AS overexpression in SCC25 cells promoted cancer cell growth, indicating the pro-growth role of HOXA10-AS transcript (Fig. 3D–F). Interestingly, HOXA10-AS mis-expression in the SCC25 cells influenced the proliferation status to a greater extent than what was observed for SAS. This phenotypic variation might be attributed to the intrinsically distinct basal expressions of HOXA10-AS among the oral cancer cells (Fig. S1), and consequently to the different extents of expression alteration.

Furthermore, we performed a xenograft experiment with the HOXA10-AS overexpressing cells and demonstrated that the HOXA10-AS overexpression promoted xenograft tumor growth, leading further support to the significance of lncRNA HOXA10-AS in in vivo cancer growth (Fig. S2). Incorporating knockdown and overexpression analyses demonstrated that lncRNA HOXA10-AS is the determinant molecule for cancer cell growth that coincided with the tumor-biased expression of HOXA10-AS transcript in various cancers (Fig. 1). The molecular interplay between the antisense transcript and the host gene delivers an additional layer of gene regulation on cancer development [23–25]. However, the expression of the parental HOXA10 and neighboring HOXA9 gene both remained unchanged during HOXA10-AS knockdown and overexpression (Fig. S3). This thereby suggests the independent regulatory mechanisms of the HOXA10 gene and its antisense transcript in oral cancer progression [26]. The separate molecular actions of lncRNA and its host gene could be attributed to distinct cellular contexts among cellular differentiation and/or cancer types.

### HOXA10-AS transcript associates with cancer cell survival and metastasis

**Fig. 4:**
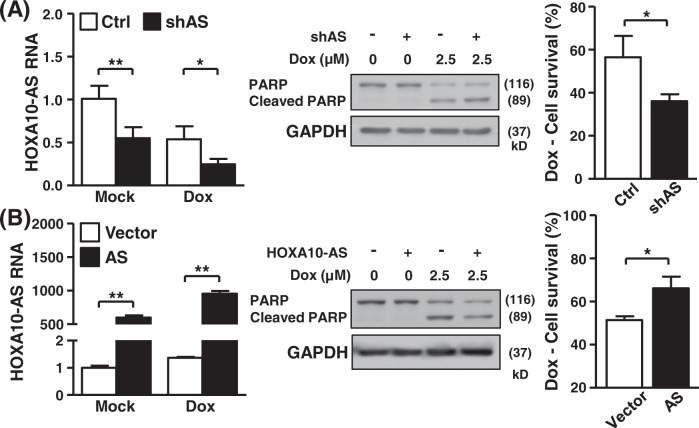
Association between HOXA10-AS expression and cancer cell survival. **A** Control and HOXA10-AS knockdown cells were treated with 2.5 µM doxorubicin to induce apoptosis. HOXA10-AS RNA level and PARP protein expression of treated cells were analyzed by RT-qPCR and Western blot assays (*n* = 3), respectively. GAPDH served as the loading control. Cell viability upon treatment was measured by MTT analysis, with normalization to the value of untreated cells. **B** Both HOXA10-AS overexpressing and control cells were treated with doxorubicin to activate cellular apoptosis. The indicated gene and protein expression of treated cells was detected by RT-qPCR and Western blot assays (*n* = 3), respectively. GAPDH expression was used as the internal control. Cell survival was assessed by MTT analysis that was normalized to the value of untreated cells.

Given that reduced cell growth may correlate with the activation of anti-death signaling, we next analyzed the effect of lncRNA HOXA10-AS on cell death response. Doxorubicin, an anticancer reagent, was used to induce cellular apoptosis. The activation of apoptosis (caspase cascade) was monitored based on PARP protein cleavage. HOXA10-AS knockdown enhanced cleaved PARP expression and decreased cell viability, suggesting the association of HOXA10-AS expression and sensitivity with anticancer treatment (Fig. 4A). Subsequently, we overexpressed HOXA10-AS in cells and analyzed cell survival under doxorubicin challenge. We observed attenuated PARP protein cleavage and increased cell viability in HOXA10-AS-overexpressing cells compared to control cells. It revealed the essential role of HOXA10-AS transcript in mediating anti-death signaling against chemotherapeutic stresses (Fig. 4B), further supporting the results of knockdown experiments. We used an additional anticancer drug, cisplatin, to monitor HOXA10-AS regulation on cellular apoptosis. Likewise, HOXA10-AS knockdown increased cisplatin-induced cell death, whereas HOXA10-AS overexpression moderated cell death (Fig. S4A, B). The combinatorial treatment with doxorubicin and cisplatin was further incorporated in knockdown and overexpression assays (Fig. S4C–F). Consequent analyses of cell survival again revealed similar results as what was observed in single-treatment experiments, thus supporting the significance of HOXA10-AS transcript in regulating the general cellular response to chemotherapeutics. These findings demonstrate the crucial role of lncRNA HOXA10-AS in regulating cancer cell survival.

**Fig. 5:**
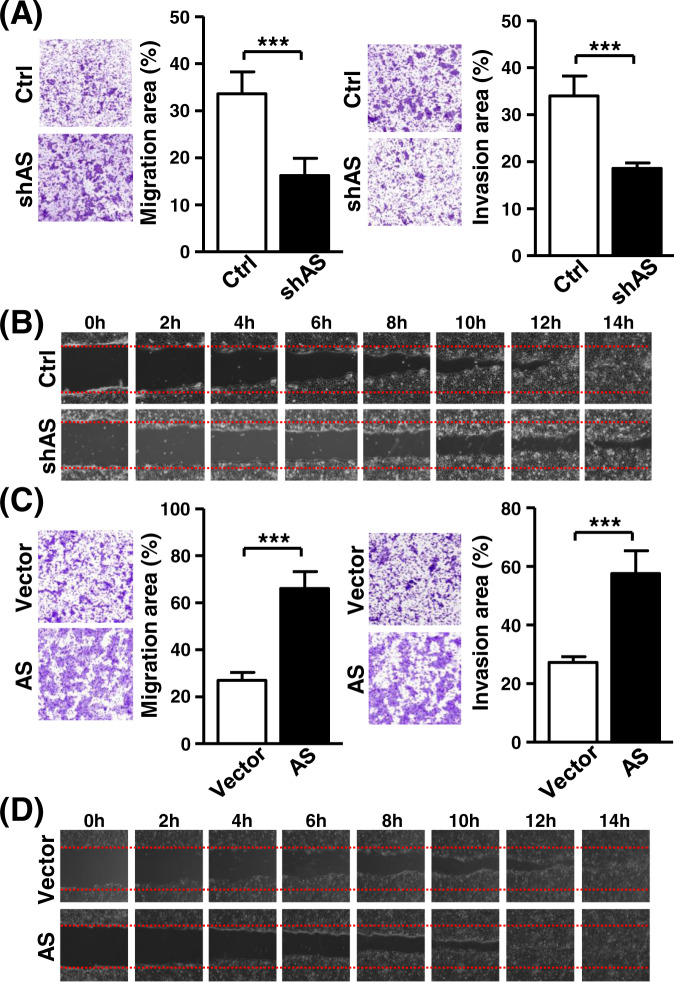
HOXA10-AS expression determines the metastatic potential of cancer cells. **A** Transwell migration (left) and Matrigel invasion (right) assays of control and HOXA10-AS knockdown cells were performed. Representative photographs are shown. The average migratory area per field was quantified and expressed in the bar graph (*n* = 3). **B** Wound-healing assay of HOXA10-AS knockdown cells was performed. Cell migration at indicated time points was recorded and shown in the representative photographs (*n* = 3). **C** Transwell migration and invasion assays of HOXA10-AS-overexpressing cells were carried out (*n* = 3). Migration and invasion abilities were quantified and shown in the bar graph. **D** HOXA10-AS-overexpressing cells were subjected to a wound-healing assay (*n* = 3). Cell migration was recorded and shown in the representative photographs.

Having established a functional connection of the HOXA10-AS transcript to cancer cell growth, we next examined its possible link to the metastasis process. To this end, cell migration capability was monitored by the wound-healing assay and Transwell-based experiments. Results suggested that the knockdown of HOXA10-AS expression abolished the migratory and invasive properties of cancer cells (Fig. 5A, B). Conversely, HOXA10-AS overexpression markedly promoted cell migration compared to the control group (Fig. 5C, D). The assays thereby illustrated the correlation of the HOXA10-AS transcript to the metastatic potential of cancer cells. Together with the growth regulatory effect, these observations functionally link HOXA10-AS upregulation to both cancer development and progression.

### Transcriptome-wide exploration of HOXA10-AS regulatory network

**Fig. 6:**
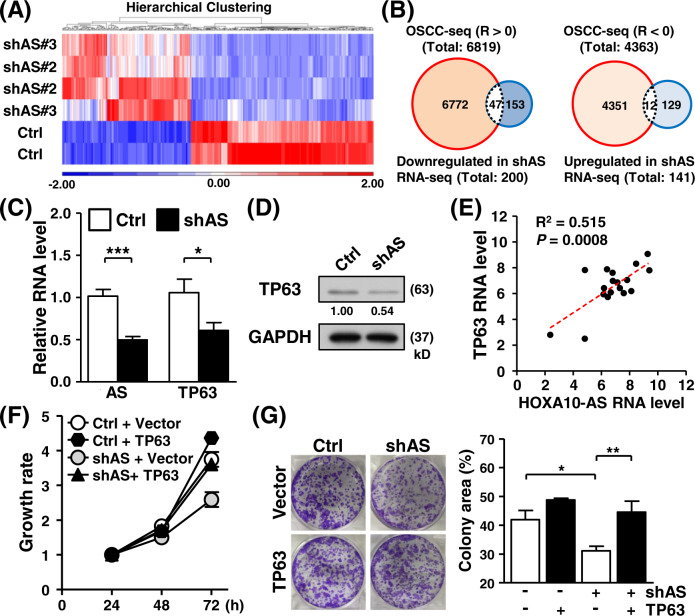
Transcriptome-wide exploration of the HOXA10-AS regulatory network. **A** Transcriptome-wide changes in HOXA10-AS knockdown cells were profiled by an RNA-sequencing assay. The differentially expressed gene profiles in the control vs. knockdown comparison were illustrated in a heatmap representation, and the expression values are displayed according to the color scale bar. **B** Venn diagrams illustrate the degree of overlap between the expressed HOXA10-AS-correlated genes from OSCC specimen RNA-sequencing (OSCC-seq) and those differentially expressed in the HOXA10-AS knockdown experiments. Forty-seven positively-correlated genes (*R* > 0) intersected with downregulated DEGs in the HOXA10-AS knockdown (left), whereas 12 inversely correlated genes (*R* < 0) intersected with upregulated DEGs. **C**, **D** The expression of the indicated genes and proteins from control and HOXA10-AS knockdown cells was measured by RT-qPCR and Western blot (*n* = 4), respectively. **E** RT-qPCR results for HOXA10-AS and TP63 mRNA expression in the OSCC cohort were subjected to a co-expression analysis (*n* = 18). Linear regression analysis was performed, and both the R-squared value and *P* value were annotated. **F**, **G** TP63 expression vector was delivered into control and HOXA10-AS knockdown cells. The transfected cells were then subjected to cell proliferation and colony formation experiments (*n* = 3).

Given that lncRNAs have been implicated in the gene regulation of diverse processes, we inspected the effects of HOXA10-AS transcript on cancer-associated pathways. RNA-sequencing assays were applied to explore transcriptome-wide alterations between control and HOXA10-AS knockdown cells. We identified 341 differentiated expressed genes (|fold change| ≥1.5, *p* value <0.05), profiles of which are depicted in a heatmap representation (Fig. 6A). We next sought to effectively identify targets of HOXA10-AS regulation through an integrative analysis of two RNA-seq datasets: the tumor specimens OSCC-seq and HOXA10-AS knockdown RNA-sequencing data. To this end, gene co-expression analyses were first done to uncover from OSCC-seq any genes in tumor samples with expression patterns positively (*R* > 0, 6819 genes) or negatively (*R* < 0, 4363 genes) correlated with HOXA10-AS. Next, the differentially expressed gene set compiled from HOXA10-AS knockdown RNA-sequencing revealed 200 downregulated genes (thus HOXA10-AS positively-correlated) and 141 upregulated genes (HOXA10-AS negatively-correlated) upon HOXA10-AS depletion. We then intersected the co-expression profiles and differential expression gene sets and subsequently identified 47 positively regulated and 12 negatively regulated genes that could be targeted by HOXA10-AS in OSCC (Figs. 6B and S5A). Ingenuity pathway analysis of the gene set revealed the possibility of the TP63 signaling pathway as a target of the HOXA10-AS regulatory mechanism [27, 28] (Fig. S5B).

Downregulated TP63 expression in HOXA10-AS knockdown cells revealed the potential of the AS-TP63 axis in regulating cancer growth (Fig. 6C, D). Further, HOXA10-AS overexpression enhanced TP63 gene expression (Fig. S5C). RT-qPCR analysis of clinical samples supported the co-expression pattern of HOXA10-AS and TP63 genes (Fig. 6E). Based on these lines of evidence, we hypothesized that TP63 potentially mediates HOXA10-AS growth regulation. To test this, a TP63 expression vector was delivered into HOXA10-AS knockdown cells. RT-qPCR and Western blot assays confirmed the specific gene expression of transfected cells (Fig. S5D, E). Functional assays showed that ectopic TP63 expression relieved the inhibited SAS cancer cell growth by HOXA10-AS knockdown (Fig. 6F, G), indicating the involvement of TP63 in HOXA10-AS regulatory signaling. Rescuing TP63 expression in SCC25 cells showed similar results, further supporting the involvement of the HOXA10-AS/TP63 axis in oral cancer progression (Fig. S6A–C). In addition, xenograft experiments using cells with rescued TP63 expression was carried out, which also corroborated the results of the cell-based assays for the functional axis (Fig. S6D). Likewise, TP63 overexpression moderated doxorubicin-induced cell death and rescued the impeded cell migration by HOXA10-AS knockdown (Fig. S7A, B). These findings collectively unravel the biological consequences of the TP63 gene in HOXA10-AS mediated oncogenic regulatory mechanisms.

### LncRNA HOXA10-AS regulates TP63 expression through scaffolding activity

**Fig. 7:**
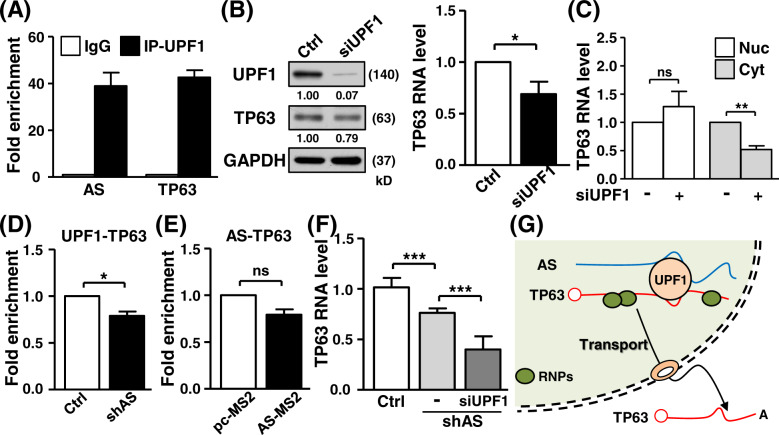
The HOXA10-AS transcript serves as a modular scaffold for TP63 RNA-processing. **A** An RNA-IP assay was performed with control IgG and UPF1-specific antibodies. The precipitated RNA was analyzed by RT-qPCR with the specific primers (*n* = 3). **B** Control and UPF1-specific siRNA were delivered into cells, and the expression of the indicated protein and mRNA of transfected cells was measured by Western blot and RT-qPCR (*n* = 3), respectively. GAPDH expression served as the loading control. **C** UPF1 knockdown cells were collected and subjected to a fractionation assay. TP63 gene expression in fractions was detected by RT-qPCR (*n* = 5). **D** An RNA-IP assay of the indicated cells was performed with UPF1-specific antibody, and the precipitated RNA was subjected to RT-qPCR assay (*n* = 4). **E** MS2-based RNA-IP assay of the indicated transfected cells was performed, and the precipitated RNA was subjected to RT-qPCR assay with specific primers (*n* = 3). **F** UPF1 siRNA was transfected into knockdown cells, and TP63 expression of transfected cells was detected by RT-qPCR experiments (*n* = 5). **G** Schematic representation of HOXA10-AS lncRNA-mediated TP63 gene regulation is shown.

Considering that lncRNAs can sequester proteins to regulate gene expression, we next implemented MS2 hairpin-specific RNA-IP coupled with a proteomic approach to explore a potential protein interactome underlying lncRNA regulation. Using mass spectrometry analysis, the UPF1 protein, a helicase for RNA-processing, was uncovered as the potential interacting protein. The UPF1 RNA-IP assay confirmed UPF1 protein interaction with the HOXA10-AS transcript, and also revealed UPF1 binding to the TP63 transcript (Fig. 7A). Independent HOXA10-AS MS2-based IP experiments showed the precipitation of UPF1 proteins (Fig. S8A), which further confirmed an interaction between UPF1 protein and HOXA10-AS RNA. Knockdown of UPF1 expression resulted in a reduction of TP63 gene expression (Fig. 7B), particularly the cytosolic expressed TP63 mRNA (Fig. 7C). Nuclear expression of the UPF1 protein uncovered by a fractionation assay supported the idea of its RNA-processing activity (Fig. S8B). These observations indicate a coordinated UPF1–TP63 regulation that is mediated by the subcellular distribution mechanism [29, 30].

Based on these observations, we next inspected the association of HOXA10-AS with UPF1–TP63 gene regulation. RNA-IP assays first showed that the interaction between UPF1 protein and TP63 mRNA was attenuated by HOXA10-AS knockdown (Fig. 7D), revealing the modular scaffold function of HOXA10-AS in such protein–RNA interactions. To provide further support to the notion that HOXA10-AS regulates TP63 processing and localization, an RNA fluorescence in situ hybridization assay was performed to demonstrate the subcellular distribution of the TP63 transcript (Fig. S8C). We subsequently detected the nuclear accumulation of TP63 RNA upon HOXA10-AS knockdown. This spatial change served as a strong evidence for the regulation of TP63 mRNA processing by lncRNA HOXA10-AS, and its connection to oral cancer malignancy. Furthermore, TP63 mRNA was not precipitated in MS2-labeling HOXA10-AS specific RNA-IP assays (Fig. 7E). This result indicated that UPF1–TP63 interactions mediated by HOXA10-AS transcript was independent of RNA–RNA mechanisms. Incidentally, a UPF1-specific knockdown in HOXA10-AS suppressed cells brought a more negative effect on TP63 expression than the control group (Fig. 7F), implying that UPF1–TP63 regulation could operate independently of the association of the HOXA10-AS transcript. These findings are in line with the notion that HOXA10-AS lncRNA establishes the recognition of the TP63 transcript by UPF1 protein, which determines the subcellular transportation mechanism underlying TP63 gene regulation (Fig. 7G).

## Discussion

We utilized extensive transcriptome profiling to identify the cancer-associated lncRNA, HOXA10-AS transcript, and subsequently performed functional assays to describe the significance of this lncRNA in oral cancer development and progression. HOXA10-AS expression has been implicated in cell survival against drug treatment. Mechanistically, we characterized the modular scaffolding function of the HOXA10-AS transcript for the binding of the TP63 mRNA by the UPF1 protein. Such action determines the subsequent transcript processing and functional consequences of cancer growth regulation. These systematic analyses and biochemical experiments delineate the molecular role of lncRNA and its mechanistic outcomes for oncogenic activation. Taken together, our results illustrate how lncRNA-mediated posttranscriptional regulation shapes the cancer transcriptome.

Recent reports have indicated the pivotal role of HOXA10-AS transcript in the progression of oral carcinoma [31, 32], the underlying mechanisms of which are associated with miRNA sponge activity. Accordingly, we attempted to inspect the possibility of lncRNA-mediated posttranscriptional regulation on TP63 expression. AGO2-specific RNA-IP assay affirmed the association of HOXA10-AS with RISC activity (Fig. S9A). In silico predictions [33, 34] discovered the likely sponged miRNAs that targeted the TP63 gene (Fig. S9B). However, ectopic expression of target miRNAs (i.e., miR-511 and miR-855) showed a negligible effect on TP63 expression (Fig. S9C). Consequently, we proposed that the HOXA10-AS transcript primarily regulates the TP63 gene in a posttranscriptional manner but not through a miRNA sponge mechanism. Such regulation by lncRNA scaffolding activity fine-tunes RNA-processing mechanisms associated with tumor survival fitness.

Current findings reveal that lncRNA HOXA10-AS-mediated protein recruitment serves as the structural scaffold and/or orientation for the consequent regulatory process. This identifies a transcriptional modifier role of lncRNA in RNA-processing and transportation mechanisms. In this respect, RNA–RNA interactions between HOXA10-AS and TP63 transcripts was not detected by the MS2-based RNA-IP assay (Fig. 7E). It alternatively suggests that lncRNA-mediated protein sequestering might provide the favorable configuration for UPF1 recognition and ensuing processing. This consequently determines the subcellular RNA distribution and the underlying biological function. In line with this hypothesis, HOXA10-AS knockdown disrupts the UPF1–TP63 protein–RNA interaction (Fig. 7D), supporting the molecular role of lncRNA scaffolding in this transcriptomic regulation. Finally, UPF1 protein expression remained unchanged upon HOXA10-AS knockdown or overexpression, excluding the possibility that UPF1 expression is under the control of lncRNA HOXA10-AS (Fig. S10). These findings together illustrate the molecular interactions and functional outcomes of lncRNA-mediated posttranscriptional regulation, providing valuable insights into the role of lncRNAs in cancer biology.

### Reporting summary

Further information on research design is available in the Nature Research Reporting Summary linked to this article.

## Supplementary information

Supplemental information

Supplemental Table S1

Reporting summary

## Data Availability

The published article includes all datasets generated/analyzed for this study.
