# Supplementary material for: Modular scaffolding by lncRNA HOXA10-AS promotes oral cancer progression
Source: Cell Death Dis. 2022 Jul 20;13(7):629. doi: 10.1038/s41419-022-05071-6 (PMC9300705; doi:10.1038/s41419-022-05071-6)

Supplemental Information

Modular Scaffolding by LncRNA HOXA10-AS Promotes Oral Cancer Progression

Yi-Tung Chen[1,2], Chia-Hua Kan[3], Hsuan Liu[1,4,5,6], Yu-Hao Liu[4], Chih-Ching Wu[2,5,7,8], Yu-Ping Kuo[1], Ian Yi-Feng Chang[1,9], Kai-Ping Chang[1,8], Jau-Song Yu[1,4,5], and Bertrand Chin-Ming Tan[2,3,5,9,]*

[1]Molecular Medicine Research Center, Chang Gung University, Taoyuan 333, Taiwan
[2]Research Center for Emerging Viral Infections, Chang Gung University, Taoyuan 333, Taiwan
[3]Department of Biomedical Sciences, College of Medicine, Chang Gung University, Taoyuan 333, Taiwan
[4]Department of Cell and Molecular Biology, College of Medicine, Chang Gung University, Taoyuan 333, Taiwan
[5]Graduate Institute of Biomedical Sciences, College of Medicine, Chang Gung University, Taoyuan 333, Taiwan
[6]Division of Colon and Rectal Surgery, Lin-Kou Medical Center, Chang Gung Memorial Hospital, Taoyuan 333, Taiwan
[7]Department of Medical Biotechnology and Laboratory Science, College of Medicine, Chang Gung University, Taoyuan, Taiwan
[8]Department of Otolaryngology-Head & Neck Surgery, Lin-Kou Medical Center, Chang Gung Memorial Hospital, Taoyuan 333, Taiwan
[9]Department of Neurosurgery, Lin-Kou Medical Center, Chang Gung Memorial Hospital, Taoyuan 333, Taiwan

*Correspondence: btan@mail.cgu.edu.tw; Tel.: +886-3-211-8800; Fax: +866-3-211-8700

Materials and Methods

Sequencing Data Analysis and Bioinformatics Analysis

The processed RNA-seq data were transferred to Partek Genomics Suite and mapped for ncRNA or mRNA annotations on the basis of GENCODE v25. Partek Genomics Suite and statistical packages were used to perform statistical analysis, hierarchical clustering, ncRNA identification, differential expression analysis, and

pathway enrichment. The ncRNA data were also integrated with the TCGA dataset to pinpoint specific ncRNA candidates/signatures, which were further selected for PCR-based verification.

RNA-Sequencing and Gene Expression Analysis

TRIzol reagent (Invitrogen, CA, USA) was used for RNA extraction, and cDNA libraries were prepared based on the TruSeq® Stranded Total RNA Sample Preparation Guide (Illumina, Part # 15031048, San Diego, CA, USA). Equal concentrations of each library were sequenced using a NextSeq 500 (Illumina) platform to create pair-end 75-bp reads. Quality assessment and trimming of the generated sequences were done by the RNA-seq alignment tool from BaseSpace (Illumina), followed by alignment to the human reference genome (hg38) with STAR 2.5.2b (1). The expression levels of genes in each sample and the corresponding fold changes were estimated by DESeq2 1.14.1 (Partek Genomics Suite, St. Louis, MO, USA) (2) with GENCODE V19 (V25) annotation (3). Relative expression of each gene is represented by CPM (Counts Per kilobase Million). Partek Genomics Suite and statistical package were used for the statistical analysis, hierarchical clustering differential expression analysis, and Gene ontology (GO) enrichment. Canonical pathway analysis was performed by using the Ingenuity Pathway Analysis (IPA).

Table S1. List of primers used in this study.

Please see the attached file.

Reference

1. Dobin A, Davis CA, Schlesinger F, Drenkow J, Zaleski C, Jha S, et al. STAR: ultrafast universal RNA-seq aligner. Bioinformatics. 2013 Jan 01;29(1):15-21. PubMed PMID: 23104886. Pubmed Central PMCID: 3530905.
2. Love MI, Huber W, Anders S. Moderated estimation of fold change and dispersion for RNA-seq data with DESeq2. Genome biology. 2014;15(12):550. PubMed PMID: 25516281. Pubmed Central PMCID: 4302049.
3. Harrow J, Frankish A, Gonzalez JM, Tapanari E, Diekhans M, Kokocinski F, et al. GENCODE: the reference human genome annotation for The ENCODE Project. Genome research. 2012 Sep;22(9):1760-74. PubMed PMID: 22955987. Pubmed Central PMCID: 3431492.

Supplemental Figures & Legends

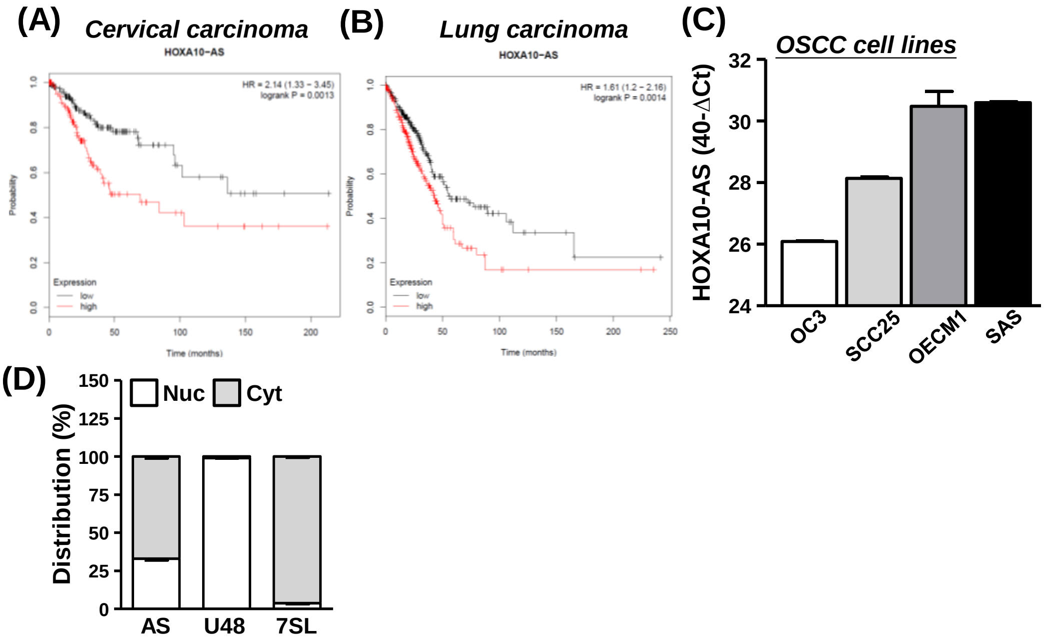

Figure S1. Association of HOXA10-AS expression with patient survival and OSCC cancer lines. (A and B) Kaplan-Meier analysis of patient survival outcome based on stratification by HOXA10-AS expression was performed usingpublic database (http://kmplot.com/analysis/index.php?p=background). (C) HOXA10-AS expression in the indicated oral cancer cell lines was assessed by RT-qPCR experiment and presented by the ΔCt method (n = 3). (D) SAS cells were collected and subjected to a fractionation assay. The indicated gene expression in fractions was detected by RT-qPCR assay (n = 3).

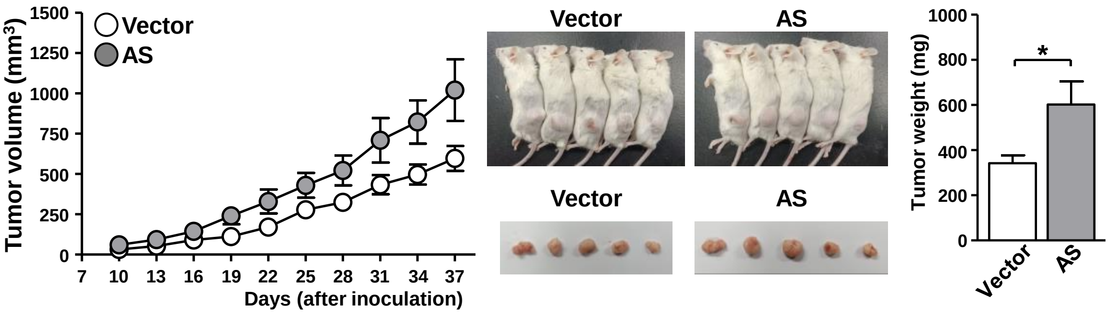

Figure S2. HOXA10-AS overexpression promotes to oral cancer growth. Mouse xenograft experiments were performed by inoculating control (Vector) and HOXA10-AS overexpressing SAS cells (AS). Tumors formed at the indicated time points were dissected and their volumes measured. The middle panel shows photographs of mice bearing tumors as well as surgically removed tumors. The bar graph depicts the average weights of the tumor masses dissected from the indicated groups at sacrifice.

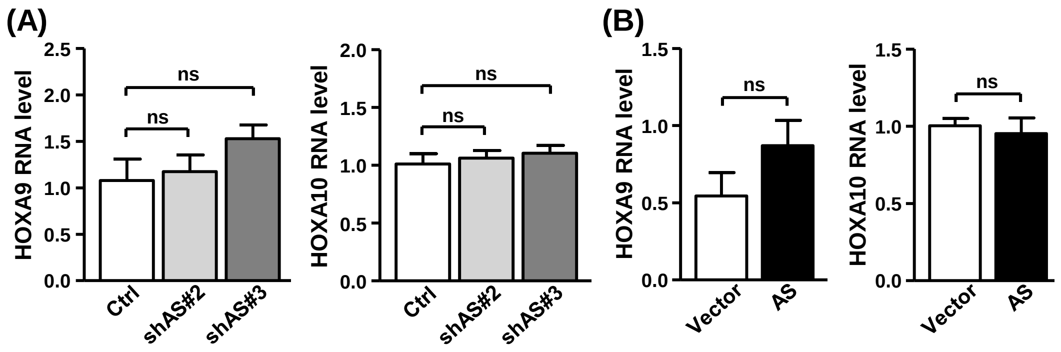

Figure S3. HOXA9 and HOXA10 gene expression was not affected at HOXA10-AS knockdown and overexpression. (A and B) Expression levels of the HOXA9 and HOXA10 gene in cells with HOXA10-AS knockdown (A) or overexpression (B) was detected by RT-qPCR assay (n = 4). For statistical analyses shown in this figure: ns P>0.05; * P<0.05.

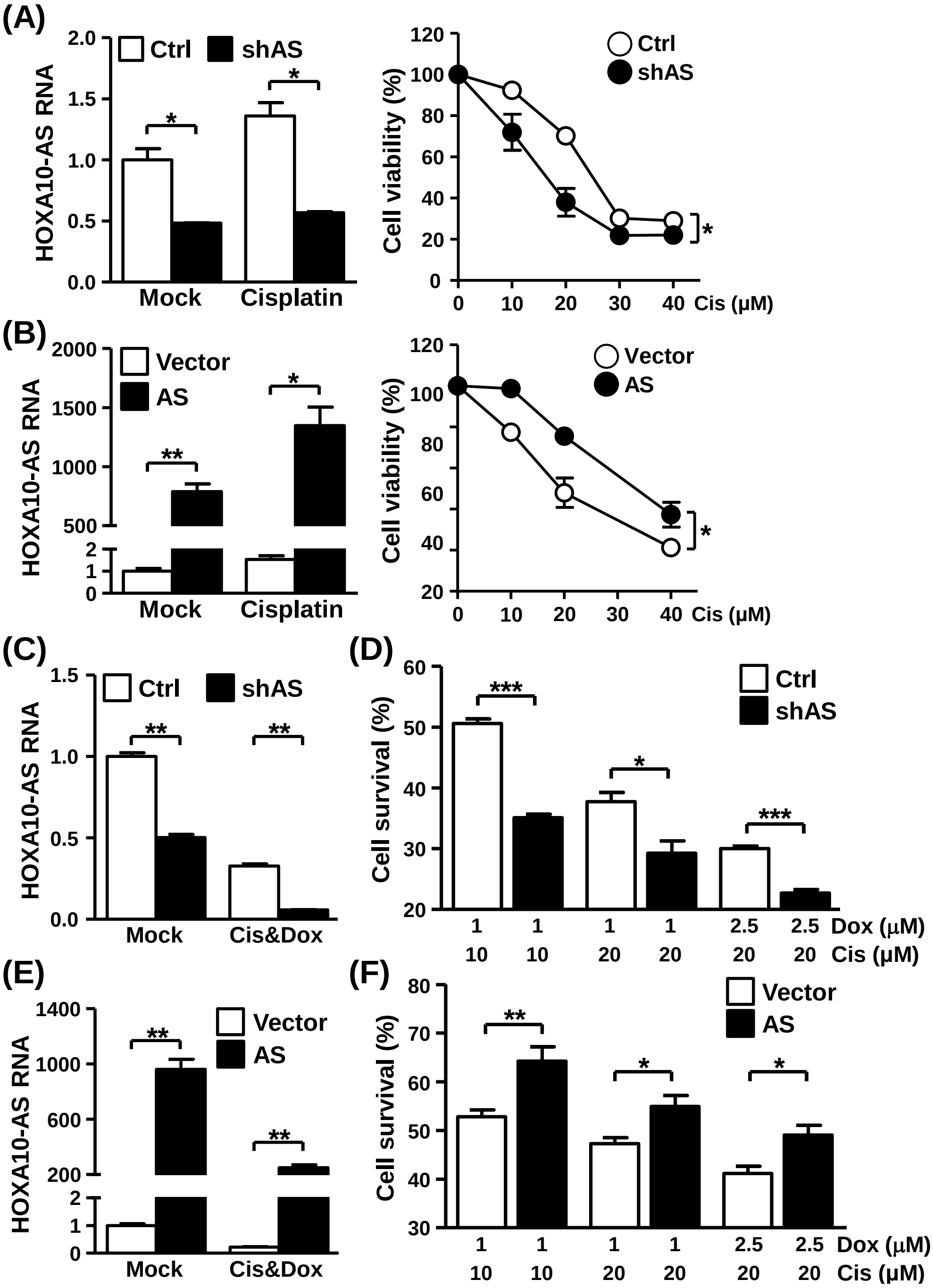

Figure S4. HOXA10-AS expression associated with cell survival upon cisplatin treatment. (A) Control and HOXA10-AS knockdown (shAS) cells were treated with the indicated concentration of cisplatin to induce apoptosis. HOXA10-AS expression of the treated cell was measured by RT-qPCR assay (left panel), and the cell survival of treated cells was monitored by MTT assay (n = 3) (right panel). (B) Control and HOXA10-AS overexpressing cells were treated with cisplatin to induce cell death, and target gene expression and cell viability of treated cells was analyzed by RT-qPCR and MTT assays (n = 3), respectively. (C) Control and HOXA10-AS knockdown (shAS) cells were treated with the 10 μM cisplatin and 1 μM doxorubicin,

and HOXA10-AS expression of the cells was analyzed by RT-qPCR (n = 3). (D) Control and knockdown cells were treated with the indicated concentration of drugs, and the cell survival of treated cells was monitored by MTT assay (n = 3). (E) Control and HOXA10-AS overexpressing cells were treated with 10 μM cisplatin and 1 μM doxorubicin, and HOXA10-AS expression of the treated cells was measured by RT-qPCR assays (n = 3). (F) Control and HOXA10-AS overexpressing cells were treated with the indicated drug concentration to induce cell death, and the cell viability of treated cells was analyzed by MTT assay (n = 3).

(A)

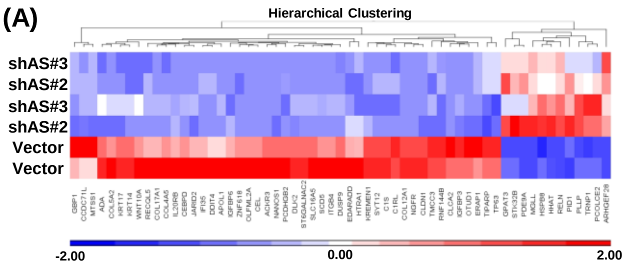

(B)

Upstream Regulator	Expr Fold Change	Molecule Type	Activation z-score	P-value	Target Molecules in Dataset
TP73		transcription regulator	-1.686	1.30×10^{-6}	ADA, COL5A2, DDIT4,HSPB8, IGFBP3, ITGB4, RELN, RNF144B, WNT10A
OSM		cytokine	-1.762	2.17×10^{-6}	C1S, CEBPD, GBP1, IFI35, IGFBP3, IGFBP6,KRT17, MGLL, OLFML2A, PLLP
lipopolysaccharide		chemical drug	-3.197	2.24×10^{-6}	ACKR3, ADA, CEBPD, CLDN1, COL12A1, COL5A2, DDIT4, GBP1,HTRA1, IFI35, IGFBP3, IL20RB, MTSS1, NGFR, PCOLCE2, PID1,TIPARP,TP63
PRDM1		transcription regulator	1.23	2.56×10^{-6}	ADA, CLDN1, DDIT4, ERAP1, GPAT3, RELN, SLC16A5
TP63	-2.332	transcription regulator	-0.857	4.39×10^{-6}	ADA, DDIT4, HTRA1, IGFBP3, IGFBP6, ITGB4, KRT14, RELN, TP63

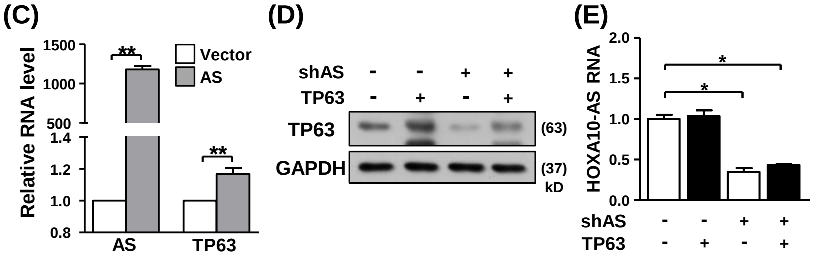

Figure S5. Heatmap representation of differential expressed gene set of HOXA10-AS knockdown cells. (A) The distribution of the expression profiles of the 59 intersected genes uncovered in OSCC-sequencing and HOXA10-AS knockdown RNA-sequencing assays was illustrated in a heatmap representation. (B) Upstream regulator for the gene set (59 genes) was analyzed by Ingenuity Pathway Analysis software. (C) Indicated gene expression in the control and HOXA10-AS overexpressing cells was detected by RT-qPCR assay (n = 3). (D and E) Specific protein and gene expression of the indicated cells (in Fig. 6F) was analyzed by Western blot and RT-qPCR assays (n = 3), respectively. GAPDH expression is measured as the internal control.

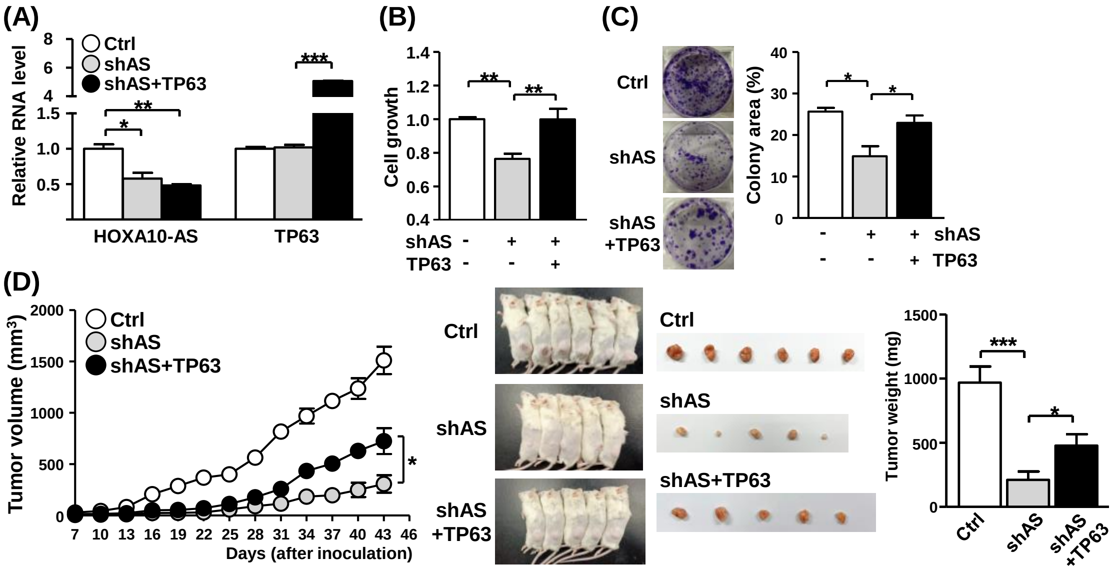

Figure S6. Ectopic TP63 expression relieved the inhibited growth of the HOXA10-AS knockdown cells. (A to C) TP63 expression vector was delivered into HOXA10-AS knockdown SCC25 cells, and the indicated gene expression of the transfected cells was analyzed by RT-qPCR assay (A). The cell proliferation rate and colony-forming ability of the transfected cells was assessed by the MTT method and crystal violet staining, respectively (n = 3). (D) Mouse xenograft experiment was performed by inoculating the control (Ctrl), HOXA10-AS knockdown (shAS), and TP63 rescuing (shAS+TP63) cells. Tumors formed at the indicated time points were dissected and their volumes measured. The middle panel shows photographs of mice bearing tumors as well as surgically removed tumors. The bar graph depicts the average weights of the tumor masses dissected at sacrifice from the indicated groups.

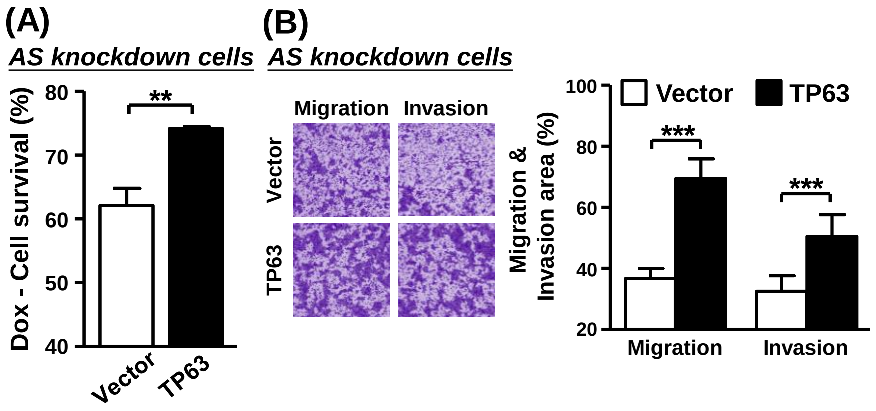

Figure S7. Rescuing TP63 expression in HOXA10-AS knockdown cells enhanced cell survival and migration ability. (A) Control and TP63-overexpressing HOXA10-AS knockdown cells were treated with doxorubicin to induce apoptosis, and cell survival upon treatment was measured by MTT assay (n = 3). (B) TP63 expression and control vectors were transfected into HOXA10-AS knockdown cells, and the migration and invasion abilities of transfected cells was analyzed by Transwell experiments (n = 3). The representative images for the migrating and invading cells is shown in the left panel, and the quantification of the results is displayed in bar graphs (right panel).

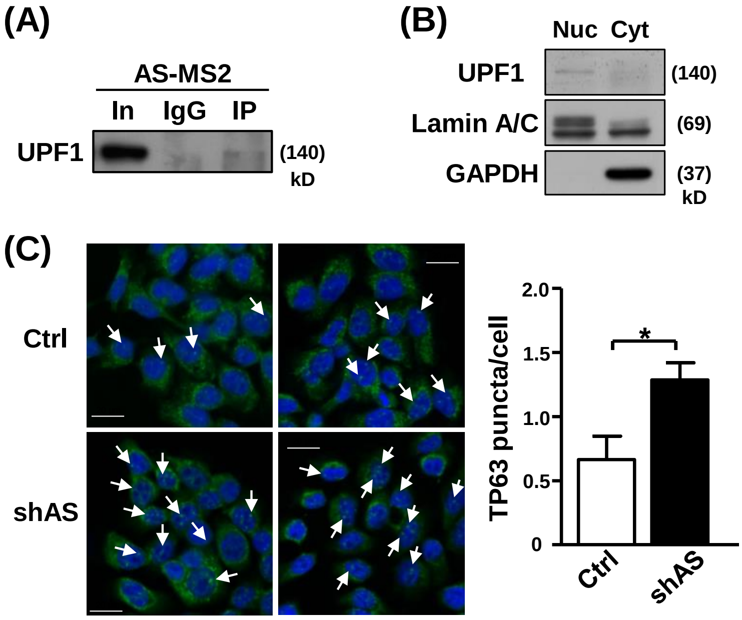

Figure S8. MS2-tagged RNA-IP assay revealed the interaction of UPF1 protein with HOXA10-AS transcript. (A) MS2-tagged HOXA10-AS expression vector (AS-MS2) was constructed and delivered into cells with Flag-tagged MS2-binding protein expression vector, and the transfected cell lysate was subjected to IP assay with control IgG (IgG) and Flag antibody (IP). The precipitates were collected and analyzed by Western blot assay, with the input (In) as the positive control. (B) Nuclear (Nuc) and cytosolic (Cyt) compartments were isolated by fractionation assay, and the indicated protein expression of fractions was analyzed by Western blot assay. (C) Fluorescence in situ hybridization of HOXA10-AS knockdown cells was performed with the TP63-specific probe (green), and nuclei were counter-stained by Hoechst 33342 dye (Blue). The arrows indicate the accumulated TP63 RNA signals in the nucleus, and the quantification of TP63 nuclear puncta per cells is shown by a bar graph in right panel (n=160).

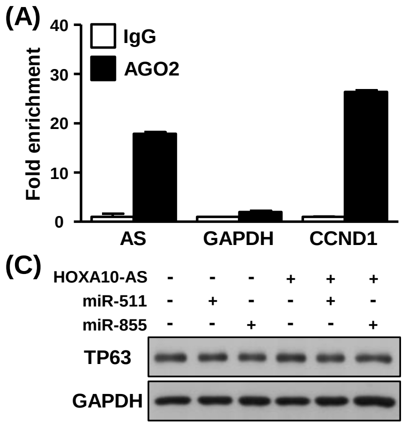

(B)

	Sponge prediction	TargetScan 7.1
**miR-511-5p**	5	1
**miR-762**	4	1
**miR-855-3p**	3	2
**miR-505-5p**	3	1
**miR-29b-2-5p**	3	1
	AS binding site	miRNA binding to TP63

Figure S9. LncRNA HOXA10-AS regulates TP63 expression in a miRNA-independent mechanism. (A) RNA-IP assay was performed with AGO2-specific antibody and control IgG, and the indicated gene expression of precipitated complex was analyzed by RT-qPCR experiment. GAPDH and CCND1 abundance in the precipitates was measured as experimental control. (B) Bioinformatics analyses for the miRNA sponge activity of HOXA10-AS transcript (sponge prediction) and miRNA prediction for TP63 gene targeting (TargetScan 7.1). (C) Candidate synthetic miRNAs were transfected into control and HOXA10-AS overexpressing cells, and TP63 protein expression of transfected cells was analyzed by Western blot assay. GAPDH expression serves the loading control.

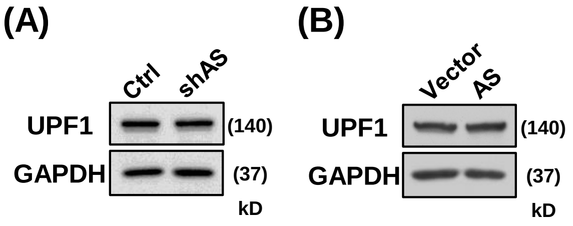

Figure S10. UPF1 protein expression remains unchanged upon lncRNA HOXA10-AS knockdown or overexpression. (A and B) UPF1 protein expression of the HOXA10-AS knockdown and overexpression cells was analyzed by Western blot assay. GAPDH expression is shown as the internal control.

Original western blots

Fig.4A

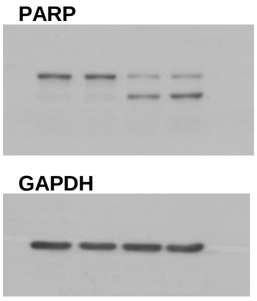

Fig.4B

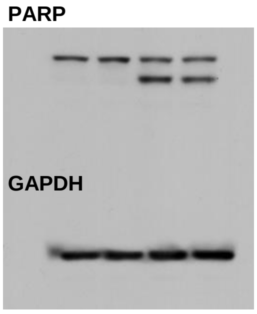

Fig.6D

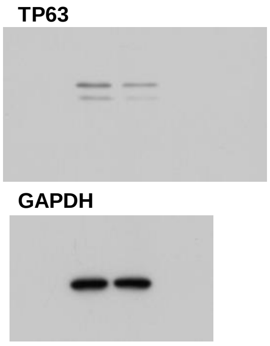

Fig.7B

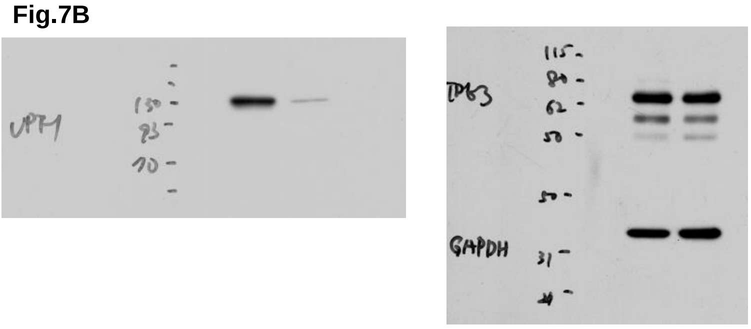

Fig.S5D

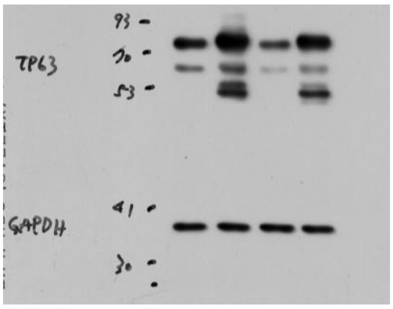

Fig.S8A

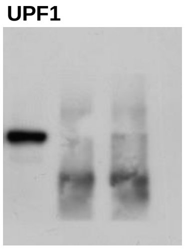

Fig.S8B

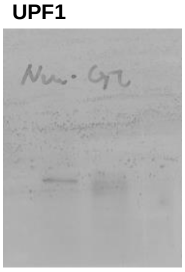

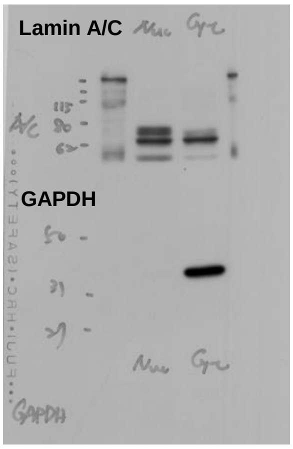

Fig.S9

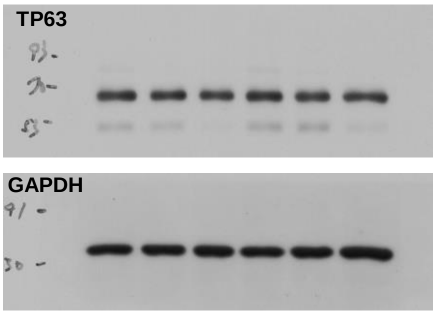

Fig.S10A

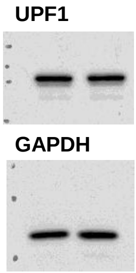

Fig.S10B

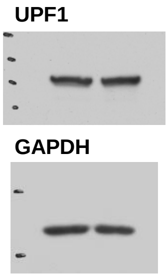

Supplement: Supplementary file 1 — Supplemental information [file 41419_2022_5071_MOESM1_ESM.pdf]
